# gbtools: Interactive Visualization of Metagenome Bins in R

**DOI:** 10.3389/fmicb.2015.01451

**Published:** 2015-12-18

**Authors:** Brandon K. B. Seah, Harald R. Gruber-Vodicka

**Affiliations:** Department of Symbiosis, Max Planck Institute for Marine MicrobiologyBremen, Germany

**Keywords:** metagenomics, exploratory data analysis, visualization, microbiology, symbiosis, binning

## Abstract

Improvements in DNA sequencing technology have increased the amount and quality of sequences that can be obtained from metagenomic samples, making it practical to extract individual microbial genomes from metagenomic assemblies (“binning”). However, while many tools and methods exist for unsupervised binning with various statistical algorithms, there are few options for visualizing the results, even though visualization is vital to exploratory data analysis. We have developed gbtools, a software package that allows users to visualize metagenomic assemblies by plotting coverage (sequencing depth) and GC values of contigs, and also to annotate the plots with taxonomic information. Different sets of annotations, including taxonomic assignments from conserved marker genes or SSU rRNA genes, can be imported simultaneously; users can choose which annotations to plot. Bins can be manually defined from plots, or be imported from third-party binning tools and overlaid onto plots, such that results from different methods can be compared side-by-side. gbtools reports summary statistics of bins including marker gene completeness, and allows the user to add or subtract bins with each other. We illustrate some of the functions available in gbtools with two examples: the metagenome of *Olavius algarvensis*, a marine oligochaete worm that has up to five bacterial symbionts, and the metagenome of a synthetic mock community comprising 64 bacterial and archaeal strains. We show how instances of poor automated binning, sequencer GC% bias, and variation between samples can be quickly diagnosed by visualization, and demonstrate how the results from different binning tools can be combined and refined to yield manually curated bins with higher completeness. gbtools is open-source and written in R. The software package, documentation, and example data are available freely online at https://github.com/kbseah/genome-bin-tools.

## Introduction

Metagenomics originated in the field of microbial ecology as a means to look into the function of whole communities, given that most environmental microbes are resistant to cultivation (Handelsman, 2004; Kunin et al., 2008; Teeling and Glockner, 2012). By shotgun-sequencing DNA from an entire microbial community, researchers can treat the resulting metagenome as a sample from the pool of genes of the entire community, and either reconstruct a picture of their collective functional potential, or assemble and extract individual microbial genomes (“binning”). While binning of genomes from metagenomes has been done in the past with relatively low-diversity samples (Tyson et al., 2004; Woyke et al., 2006), recent advances in high-throughput sequencing have vastly increased the sequencing depth that can be obtained with the same resources, and this has made it practical to bin individual genomes from increasingly diverse communities.

Strategies for binning can be classified by the source of the information that they use for separating genomes from each other: (i) the internal statistical properties of the sequence, e.g., *k*-mer frequencies (Teeling et al., 2004), (ii) comparison to external sequence information, e.g., conserved taxonomic marker genes (Kembel et al., 2011) or entire nucleotide databases (Kumar et al., 2013), or (iii) the biological or technical variation in sequence abundance/coverage between different read libraries (the differential-coverage approach) (Albertsen et al., 2013; Imelfort et al., 2014; Nielsen et al., 2014). A variety of statistical and machine learning methods have been applied to the problem, including self-organizing maps (Dick et al., 2009), interpolated Markov models (Strous et al., 2012), expectation-maximization (Wu et al., 2014), and *k*-medioids clustering (Kang et al., 2015), with the ultimate aim of binning microbial genomes from many samples automatically and with high throughput.

Visualization is usually the first step in data exploration, and despite the sophistication of many of the current methods for unsupervised binning, it remains an important part of the metagenomics toolkit. For low-diversity, high-coverage samples, such as those encountered in host-symbiont systems, or microbial consortia, it may already be possible to define bins manually from coverage-GC plots, where read coverage is plotted against the proportion of G and C bases in the sequence (GC%) for each contig, or from differential-coverage plots, where for each contig the coverage in one read library is plotted against the coverage in another read library. Contigs originating from the same genome are expected to have similar sequence composition (represented by GC%) and abundance (represented by coverage), and so should cluster together in these plots. Each cluster therefore represents a single putative genome bin. Such a heuristic approach was used by Albertsen et al. (2013) to extract 12 nearly complete genomes of uncultivated bacteria from an activated sludge community, with the aid of principal-components analysis of tetranucleotide frequencies and additional taxonomic information from marker genes overlaid on the plots. Visualization is also useful *post hoc*, to spot potential artifacts from imperfect binning, and to verify or troubleshoot automated methods.

However, existing visualization tools are mostly attached to particular binning methods or pipelines; as such, their application is relatively narrow (**Table 1**). Albertsen et al. (2013) have made available their scripts (written in the statistical computing language R) for plotting and manual binning, but these require extensive customization for new data sets. Our motivation was therefore to provide a tool that integrates data relevant to metagenome binning and let the end-user perform data exploration and visualization in an intuitive way.

**Table 1:** Comparison of features available in visualization tools for metagenomic binning.

Feature	Blobology	MetaWatt	GroopM	gbtools
Coverage-GC plots	+	+	-	+
Differential coverage plots	-	-	+	+
Plot taxonomic annotations	+	+	-	+
Import annotations from third-party tools	-	-	-	+
Import bins from third-party tools	-	-	+	+
Merge two bins	-	+	+	+
Subtract one bin from another	-	-	+	+
Export plot graphics	+	+	+	+
Interactively select bins from plots	-	+	-	+

## Methods and Implementation

The gbtools software, documentation, and example data are available online at: https://github.com/kbseah/genome-bin-tools/. The online manual provides a multi-chapter walk-through of installation, data import, data exploration, and manual bin curation. Commands and data for reproducing the usage examples and **Figures 2–6** are given in the Supplementary Information to this paper.

### Example Workflow: Contig Annotation

The binning process begins with a metagenomic sequence assembly. Each contig or scaffold (from here onward, the term “contig” refers to both) in the assembly is then annotated with data relevant to the binning procedure; these annotations are imported into the R workspace with gbtools. The coverage value for each contig in the assembly is calculated by mapping the read library back onto the assembly, e.g., with the short-read aligner bbmap.sh, from the BBtools suite, version 34 (Bushnell, 2015). This produces a mapping file in the SAM format, from which coverage values are calculated with pileup.sh from BBtools (Bushnell, 2015), which also reports the GC% of each contig. Conserved protein-coding marker genes are identified in the assembly and assigned to taxonomic groups with Amphora2 (Wu and Scott, 2012) or Phyla-Amphora (Wang and Wu, 2013). Alternatively, an approximate phylogenetic affiliation of each contig can be obtained by Blastn alignment (Camacho et al., 2009) against the NCBI nt database, using part of the Blobology pipeline (Kumar et al., 2013). Small-subunit ribosomal RNA (SSU rRNA) genes are identified with barrnap version 0.5 (Seemann, 2015) and classified by comparison to the SILVA database version 119 (Quast et al., 2013) using Vsearch version 1.1.1 (Rognes, 2015). tRNA genes are annotated with tRNAscan-SE 1.23 (Lowe and Eddy, 1997). In principle, users may choose other software tools than the above for the read-mapping and marker gene annotation, so long as the results are formatted as text files with the appropriate column headers for input to gbtools. Wrapper scripts, example commands, and a description of each input file type are given in the package documentation.

### Integrating and Visualizing Data with gbtools

Contig annotations and coverage information are imported into an R version 3.1.1 workspace (R Core Team, 2014) for analysis with the package gbtools.

gbtools organizes the imported data as objects within the R workspace. There are two object classes, corresponding to metagenomes (the “gbt” class), and to bins defined from metagenomes (the “gbtbin” class). In this way, all the data relating to a single metagenome or a single genome bin are stored in a single object. The minimum data required to create a new gbt object are the coverage and GC% values for the contigs of a single metagenome. However, coverage data from more than one read library mapped to the same metagenome can be imported simultaneously. Similarly, more than one set of marker gene or taxonomic annotations can be stored in one gbt object, e.g., when such annotations are produced by different tools or pipelines. Functions are defined for the two object classes in gbtools to produce plots and overlays, report summary statistics, and create or manipulate bins.

Coverage-GC and differential-coverage plots can be produced with the familiar plot() function; the package provides a plot() method for the gbt class. When more than one set of coverage data are available, then the user can specify which set(s) to use for plotting. If taxonomic marker data are available, they are automatically used to color the plot. If more than one set of taxonomic markers have been imported, the user can choose which marker set to overlay on the plot, and which taxonomic level (kingdom to species) to use for coloring, in order to compare the results of different taxonomic-classification tools or pipelines. Differential coverage plots can also be colored by the GC% of each contig. Contigs with SSU rRNA and tRNA genes can be marked on the plot; if available, the taxonomic assignments of those SSU rRNA genes can be added as labels. Typing the name of a gbt or gbtbin object, or using the summary() function, gives a summary of the assembly and marker statistics, e.g., total length, N50, and how many marker genes are present.

Bins can be defined from a “parent” gbt object in several ways. New bins can be created interactively by selecting a region from a coverage-GC or differential-coverage plot of a gbt object. They can also be created by specifying cutoff values for contig length, coverage, and/or GC%. It is also possible to simply supply a shortlist of contig names. If a third-party binning tool has been used to produce a set of Fasta files each corresponding to a single bin, a wrapper script is provided with gbtools to tabulate the contig names and bin names so they can be imported by gbtools to create new gbtbin objects in the R workspace for those bins. Similarly to its parent object, typing the name of a gbtbin object reports summary statistics, which includes the number of taxonomic markers and how many of them are single-copy. Two bins can be combined into a new bin object (taking the union), or the difference between two bins can be taken (relative complement). If taxonomic marker data are supplied, then it is also possible to filter contigs in a bin by the taxon assignments of the markers, retaining only those contigs that are assigned to a certain taxon.

Bin objects can also be plotted or overlaid on existing plots with the point() function. If more than one set of coverage data are available, then the user need only change one parameter in the plotting commands to specify which set to use to generate a coverage-GC or differential-coverage plot. This provides a quick way to see how a bin “behaves” with coverage data from different samples. Multiple bins can also be overlaid onto a single plot, each in a different color.

### Usage Examples

To demonstrate the use of gbtools, we used two publicly available metagenome datasets: (1) the symbiotic oligochaete worm *Olavius algarvensis*, and (2) the synthetic community of Archaea and Bacteria from Shakya et al. (2013).

Read libraries from the *O. algarvensis* metagenome, sequenced in 2013, were downloaded from the Integrated Microbial Genomes portal [Joint Genome Institute (JGI) project IDs 1021953 and 1021959^1^, ^2^]. This species is known to harbor up to five bacterial symbionts: two Gammaproteobacteria (Gamma1 and Gamma3), two Deltaproteobacteria (Delta4 and Delta1), and a Spirochaeta (Dubilier et al., 2001; Woyke et al., 2006; Ruehland et al., 2008; Kleiner et al., 2011). Of these, the Gamma1, Gamma3, and Delta4 symbionts are the most abundant (Ruehland et al., 2008). This makes it a relatively low-diversity microbial community that should be amenable to visualization and differential-coverage binning.

The two metagenome libraries represent two separate *Olavius* host individuals. They were sequenced as 150 bp paired-end reads with the Illumina HiSeq 2000 and 2500 platforms. A subset of 15 million read-pairs from the first library were assembled with IDBA-UD (Peng et al., 2012). Coverage values of both read libraries were calculated separately by mapping onto the assembly. Contigs were taxonomically annotated with Amphora2 markers and the Blobology pipeline, while SSU rRNA genes were identified with barrnap, as described above.

Two automated binning tools were applied to the assembly, using default parameters: MetaBAT version 0.25.4 (Kang et al., 2015) and MetaWatt version 3.5 (Strous et al., 2012). Both tools use tetranucleotide frequency profiles as the main source of information to define bins. The automated binning results were parsed with a Perl script; the resulting table was imported to R and converted to gbtbin objects. Lists of contigs in the draft and curated Gamma1 bins were exported from R, for evaluation with the genome-quality tool CheckM (Parks et al., 2015) using the Gammaproteobacteria taxon-specific workflow.

The second example is a synthetic community that comprises 16 archaeal and 48 bacterial strains, which was used to compare the results of community characterization from rRNA amplicon sequencing vs. metagenomic sequencing (Shakya et al., 2013). We used the metagenomic data sequenced as 100 bp paired-end reads on the Illumina HiSeq 2000 platform, available from NCBI SRA^3^ under accession number SRX200676. The reads were filtered and trimmed to remove reads with Phred quality <10 and trimmed to remove TruSeq adapter sequences, with bbduk.sh from BBtools (Bushnell, 2015), and then assembled with Megahit under the “meta” setting (Li et al., 2015). Coverage and taxonomic markers were annotated as above. Automatic binning was performed with MetaBAT and parsed/imported to R as described above. Curated merged bins were exported from R and evaluted with CheckM using the lineage-specific workflow.

## Results and Discussion

### Design of the Software Package

**FIGURE 1:**
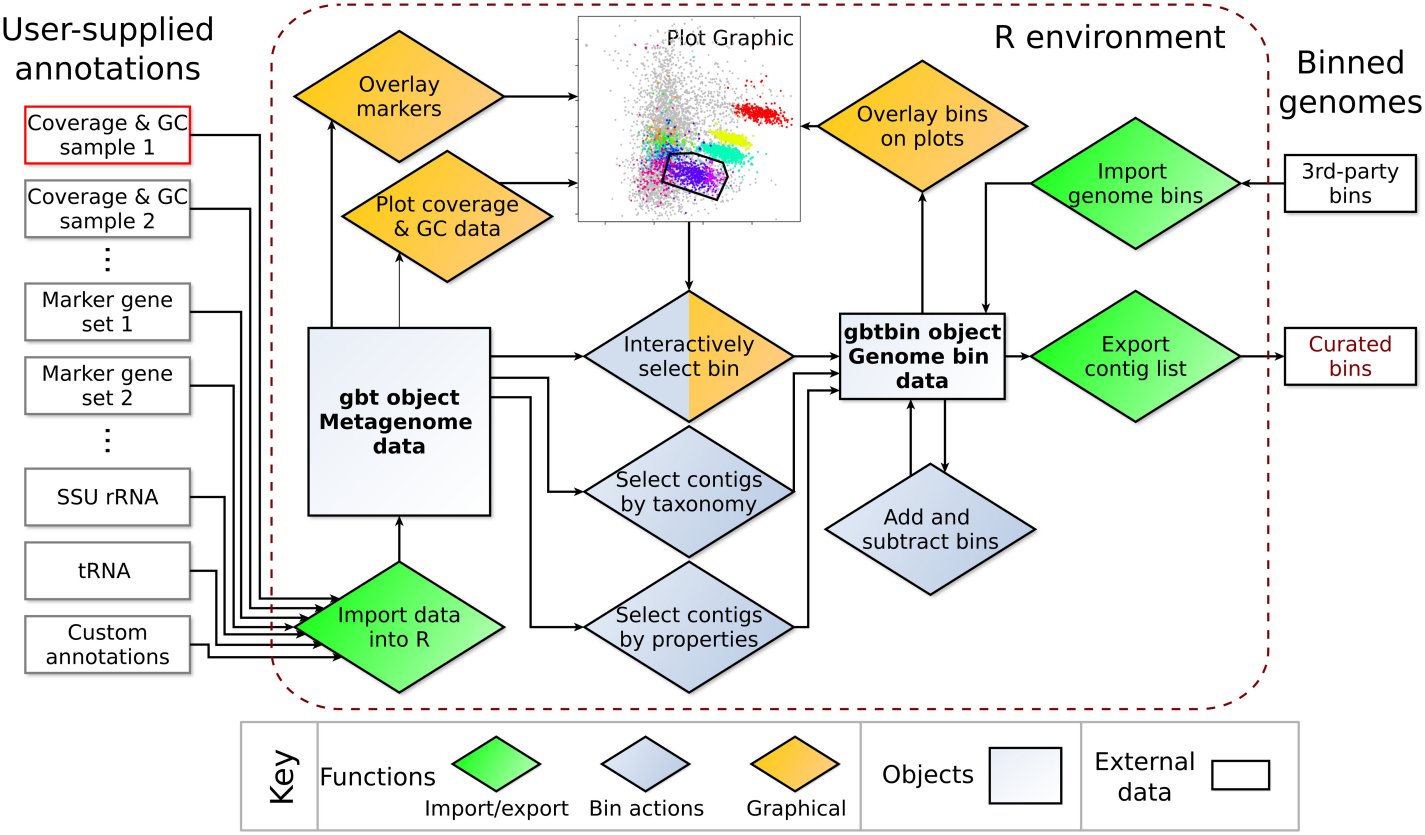
**Diagram of objects and functions available in the gbtools package.** All user-supplied annotation data associated with one metagenome (left) are integrated into a single gbt object. For each metagenome, the GC% and coverage data from at least one read library must be supplied, whereas all other input data are optional. Functions (diamonds) in the gbtools package allow import and export of data (green), visualization with plots and overlays (orange), and creating or manipulating of bins (blue).

The concepts implemented in gbtools, in particular GC-coverage and differential coverage plots, and the use of taxonomic information from marker genes (including protein-coding genes and RNA genes) to annotate these plots, are not new. What gbtools additionally offers are high-level commands for visualizing and exploring the data, “arithmetic” operations for genome bin manipulations, and an extensible, open-source framework that is amenable to future development (**Figure 1**).

Higher-level commands streamline the process of data exploration and make it more intuitive. Albertsen et al. (2013) also use R as an environment for analyzing differential coverage data, and some of the code in gbtools is adapted from their work. They provide example commands showing how functions from existing packages can be used to produce plots and perform binning. However, these commands have to be manually copied, edited, and pasted at each step, because many of the parameters for plotting have been modified from their defaults in R. gbtools conceals many of these lower-level tasks from the user, freeing up more time and attention for actually exploring the data. For example, different colored overlays for the taxonomic affiliation of contigs can be switched on and off with a single parameter in the gbtools plot() function.

A deliberate decision was made to define object classes (within the S3 object orientation system in R), rather than to create custom function names, so that the two most important tasks – drawing plots and viewing summary statistics – can be performed with the commands plot() and summary(), whose names are already familiar to most R users. Likewise, the default behavior when typing an object name is to show its summary, which displays metrics commonly used for assessing the completeness and quality of a genome, such as marker gene counts, total contig length, and contig N50.

gbtools encourages data exploration by making it possible to string together fairly complex operations like an “arithmetic” for bin manipulations. For example, one could manually define a bin from a GC-coverage plot, take a subset of those contigs above a length cutoff, extract only those contigs with marker genes classified to a certain taxon, and then see how this new bin looks like in a differential coverage plot with coverage values drawn from a different pair of samples. Intermediate steps can be saved as separate objects, so it is possible to backtrack or branch a series of operations. The command history is embedded in the bin objects themselves, so that all user actions are documented and reproducible.

Existing visualization tools tend to be limited by the specific binning tools that they were designed to complement (**Table 1**). For example: GroopM (Imelfort et al., 2014) can color contigs by GC% or by the automatically defined bins, but it does not yet support adding marker-gene based annotations; Blobsplorer (Kumar et al., 2013) shows taxon-annotated GC-coverage plots, but cannot display differential coverage plots; MetaWatt (Strous et al., 2012) is a powerful tool that implements many functions for assessing the completeness of genome bins, but the plots cannot be customized and do not show individual contigs. Being aware of this, we aimed to make gbtools as flexible as possible. For example, there have been several sets of conserved, purportedly single-copy genes published for the purpose of phylogenetic analysis or checking genome completeness (Wu and Scott, 2012; Wang and Wu, 2013; Darling et al., 2014; Parks et al., 2015). gbtools users can import the results of different marker sets together into a single gbt object, and choose which set they wish to plot as a color overlay.

By implementing gbtools in R – which is open-source, has a rich software development ecosystem, and which many scientists are already familiar with – we aimed to make it easier for users to write their own extensions for their own needs. Plots produced by gbtools can be readily exported to various formats by the native graphics engine in R, for manual editing to publication quality. Because the gbtools classes are designed to be extensible, users can import their own variables, e.g., tetranucleotide frequencies, and attach them to gbt or gbtbin objects for display or processing. Users who are already familiar with R can take advantage of its extensive statistical functions to perform additional analyses of their own, alongside graphical data exploration with gbtools.

### Usage Example: Exploration of a Challenging Assembly

The symbiotic worm *O. algarvensis* has up to five known symbiotic bacteria with different abundances, as summarized above (Ruehland et al., 2008). Nonetheless, each of them is believed to play an important biological role in this symbiosis (Woyke et al., 2006; Kleiner et al., 2012). The first published metagenome of *Olavius* (Woyke et al., 2006) used three different capillary-sequenced libraries constructed from the DNA of a total of ca. 600 animals collected at different times; binning of the symbiont genomes was based on intrinsic sequence information only (*k*-mer composition, values of *k* ≤ 6). It was necessary to pool a large number of animals because the sequencing technology at the time required milligram quantities of DNA. In contrast, the metagenome used here to illustrate the capabilities of gbtools was assembled from a single-host-animal read library sequenced on an Illumina platform. Single-host samples illustrate the inter-individual variability in relative symbiont abundance, which could be exploited for differential coverage binning.

**FIGURE 2:**
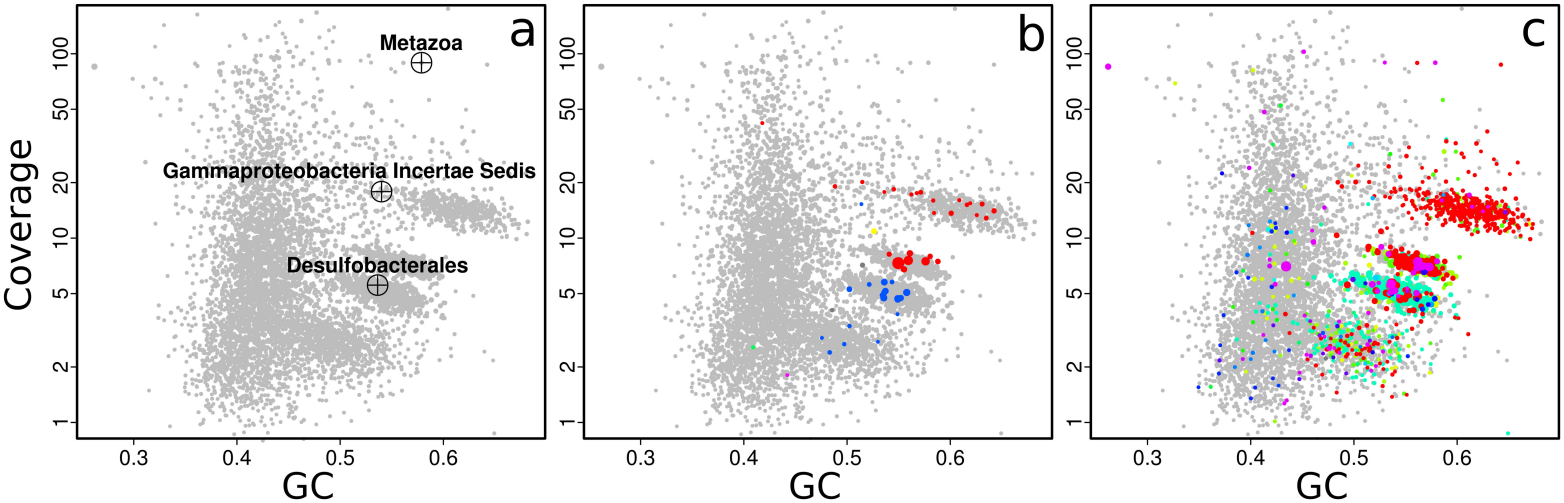
**Coverage-GC plots for an *O. algarvensis* metagenome (plot symbols are scaled by contig length), illustrating overlays for taxonomic affiliation: **(a)** crosshairs mark contigs with SSU rRNA genes, and are labeled by their affiliation in the SILVA taxonomy, **(b)** contigs containing conserved marker genes colored by taxonomic affiliation at class level (red – Gammaproteobacteria, blue – Deltaproteobacteria), **(c)** taxonomic affiliation of contigs at class level by direct Blastn search vs. the NCBI nt database (modified Blobology pipeline) (red – Gammaproteobacteria, cyan – Deltaproteobacteria, other colors – lower-abundance taxa).** Colors in **(b,c)** are arbitrary. Plots in **Figures 2–6** are identical to on-screen output, except enlarging axis labels and crosshairs for legibility, adding labels in **Figure 3c**, and adding arrows in **Figures 4b,c**.

The relative abundances of the symbionts are reflected in the metagenome used in this manuscript. Contig clusters corresponding to the Gamma1, Gamma3, and Delta4 symbiont genomes can already be seen in the plot of coverage vs. GC%, and match the taxonomic markers (single-copy conserved genes from the Amphora2 bacterial marker set, and SSU rRNA genes) overlaid on the plot (**Figure 2**). The SSU rRNA genes of the *Olavius* animal host and the Gamma1 and Delta4 symbionts were assembled and identified, but the SSU rRNA sequences of the other symbionts were not identified (**Figure 2a**). Sequences from the other symbionts could be present but have too low coverage in this assembly. The large “cloud” of relatively short contigs with GC% between 35 and 50% is probably from the host animal genome.

**FIGURE 3:**
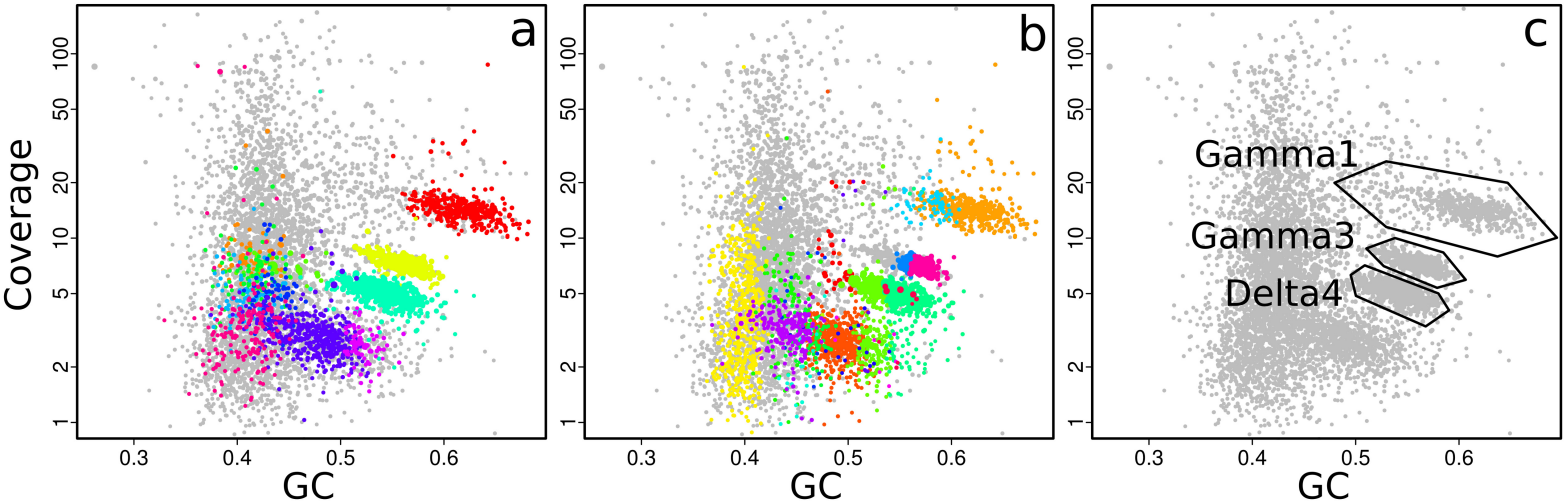
**Coverage-GC plots of an *O. algarvensis* metagenome, illustrating overlays for visualizing multiple genome bins: **(a)** 11 genome bins produced by MetaBAT, each in a different color, **(b)** 20 genome bins produced by MetaWatt, each in a different color, **(c)** boundaries of manually defined bins that were interactively selected on the plot, corresponding approximately to the indicated symbiont genomes.** Colors in **(a)** and **(b)** are arbitrary.

With gbtools, one can quickly check if apparent contig clusters may plausibly correspond to a single microbial genome. For example, the uppermost contig cluster marked in **Figure 3c** contains the 16S rRNA sequence of the Gamma1 symbiont, but the GC% values seem to have an unusually wide spread for a single microbial genome, between ca. 50 and 70%. The indicated polygon region was selected with the interactive choosebin() function, and was found to have a total length of 2.8 Mb, and contains 31 of 32 single-copy Amphora2 marker genes, all assigned to Gammaproteobacteria. However, there was additionally a single marker assigned to Deltaproteobacteria. Therefore, this cluster probably represents most of the Gamma1 symbiont genome, despite the wide GC% spread, although there is some contamination with contigs from other genomes.

The performance of different automatic binning tools can also be visually compared side-by-side. MetaBAT and MetaWatt both use tetranucleotide frequencies as the main source of information for defining bins, but apply different statistical methods to the data. The results from these two tools were parsed, imported, and used to make colored plots, where each color corresponds to a different bin; the bins predicted by MetaBAT and MetaWatt are shown in **Figures 3a,b**, respectively. The two programs produced a total of 11 and 20 bins, respectively. These plots show that MetaBAT seems to produce less-fragmented bins than MetaWatt, when default settings are used. For example, MetaBAT predicts a bin that appears to contain most of the Gamma3 symbiont genome (**Figure 3a**, yellow, with 30 of 32 Amphora2 markers), whereas MetaWatt assigns only a part of these contigs to two separate bins (**Figure 3b**, purple and dark blue, 21 and 3 markers, respectively).

However, both tools do not perform well with the genome of the primary symbiont Gamma1. MetaBAT assigns only part of the Gamma1 genome to a single bin (**Figure 3a**, red, 18 of 32 markers), whereas MetaWatt assigns fragments to two separate bins (**Figure 3b**, orange and light blue). None of these three bins contain an SSU rRNA gene. These partial bins of the Gamma1 from both MetaWatt and MetaBAT can be combined into a consensus bin with the add() function in gbtools, and were found to contain 791 contigs, with only 23 of 32 markers. Alternatively, the two MetaWatt bins can be subtracted from the MetaBAT bin with the lej() function, showing that six scaffolds with four markers were binned by MetaBAT but not MetaWatt. Such “arithmetical” operations are a natural and intuitive means of comparing the binning results. It is possible that tetranucleotide-based binning methods do not perform well with genomes that have a wide spread of GC% values, like the Gamma1. This GC% spread could possibly reflect horizontal gene transfer or different selective pressures acting on different parts of the genome.

**FIGURE 4:**
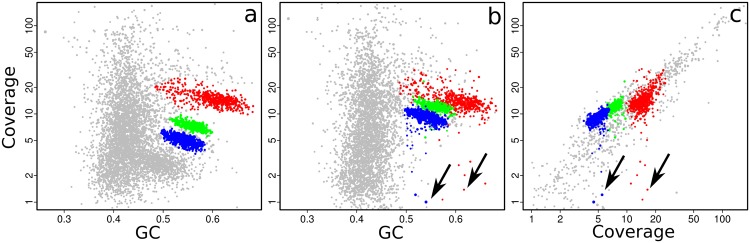
**Coverage-GC plots **(a,b)** and differential coverage plot **(c)** of an *O. algarvensis* metagenome, illustrating the appearance of manually defined genome bins (colored overlays) from **Figure 3c**: **(a)** with the original coverage data, **(b)** with coverage data from a different read library, and **(c)** when the two sets of coverage data are plotted against each other.** Contigs with GC <45% were omitted from **(c)** for clarity, as they are mostly from the host animal genome. Arrows indicate contigs which were included in the originally defined bin but have low coverage in another read library, suggesting that they may be contaminant sequences or strain variation.

The plausibility of a bin can also be tested by seeing whether its contigs still cluster together when coverage data from other samples are used. This is easily done in gbtools by varying the “slice” parameter of the plot() and points() functions. **Figure 4a** shows the three bins that were created by drawing polygons (**Figure 3c**) interactively on the coverage-GC plot to define them. The same bins form overlapping clusters when coverage data from a different read library are plotted (**Figure 4b**). Manual bin definition from a coverage-GC plot would have been less successful with that read library; in that sample, the three symbionts may have been more similar in their abundance than in the first sample. In both the coverage-GC plot of the second sample (**Figure 4b**), and the differential coverage plot of the two samples plotted together (**Figure 4c**), there are contigs with considerably lower coverage in the second sample than the first (arrows). These may represent contaminant sequences that do not actually belong to the target genome. Alternatively, they may represent genomic variation between different samples – e.g., genes that are present in some samples but not others because of inter-individual variation.

We combined the results from manual bin selection, the two automated binning tools, and differential-coverage data to produce a manually curated bin for the Gamma1 symbiont. The bin-manipulation functions in gbtools were used to perform the following actions: the interactively defined Gamma1 bin (selected from the coverage-GC plot) was merged with the partial Gamma1 bins that were produced by MetaBAT and MetaWatt. To remove likely contaminants, we removed the contig containing a Deltaproteobacteria-affiliated marker, any contigs that were in the Gamma3 or Delta4 bins produced by MetaBAT, and any contigs with <5-fold coverage in the second read library. The manual curation produced a Gamma1 bin with higher completenesss than either the interactively selected or automatically binned drafts, although the curated bin had a slightly higher contamination score (**Table 2**).

**Table 2:** Comparison of bins of the Gamma1 symbiont in the *Olavius algarvensis* metagenome, produced by interactive selection from plot, automated binning with third-party tools, and a manually curated merger and refinement of the above.

Bin source	Length (Mb)	Contigs	Amphora2 markers		CheckM completeness (%)	CheckM contamination (%)
			Total	GPB		
Interactively selected from plot	2.80	1193	32	31	90.2	0.72
MetaBAT	1.38	342	18	18	57.8	0.56
MetaWatt (merger of two bins)	2.13	785	19	19	87.7	1.12
Curated final bin	2.75	1156	31	31	94.1	1.12

### Usage Example: Visualizing a Diverse Synthetic Metagenome

The Archaea-Bacteria metagenome (from here on abbreviated as AB metagenome) (Shakya et al., 2013) represents a synthetic mock community of 64 microbial strains, an order of magnitude more diverse than the *Olavius* symbiont example described above. Because the number and phylogenetic placement of the component microbial genomes is known in advance, the AB metagenome is useful for testing the effectiveness of binning methods. Nonetheless, it is arguably less complex than a real microbial community because the strains have a wide phylogenetic distribution (close relatives can be more difficult to bin because of sequence similarity), high clonality because they come from pure cultures, and lack contaminating eukaryotic DNA which can make assembly more difficult.

**FIGURE 5:**
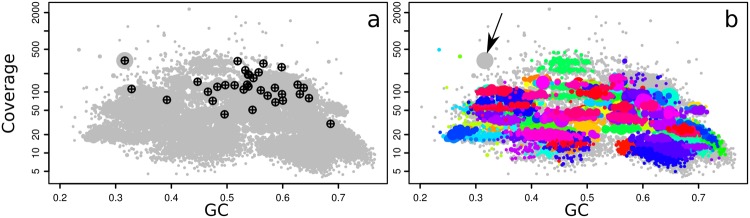
**Coverage-GC plots for the synthetic AB metagenome (plot symbols are scaled by contig length). (a)** Coverage levels show decrease at extreme GC values; note logarithmic scale in vertical axis. Crosshairs mark contigs with SSU rRNA genes, which tend to have higher coverage and more moderate GC values on average than other contigs. **(b)** Genome bins produced by MetaBAT as colored overlays (colors arbitrary), showing that not all contigs were assigned to a bin, including some high-coverage contigs; unbinned contigs shown in gray. The genome of *Nanoarchaeum equitans* assembled into a single contig (arrow) and was therefore not assigned to a bin by MetaBAT.

Visualization of metagenomic binning remains useful as a diagnostic tool, despite the higher complexity of this metagenome. The coverage-GC plot shows a pronounced hump at moderate GC values, but coverage falls off at high (>70%) and low (<30%) values (**Figure 5a**). Error-rate and coverage biases at high and low GC are known to aﬄict various sequencing platforms, potentially causing problems for assembly and downstream analyses (Ross et al., 2013). However, there is no obvious indication from the plot that the AB metagenome assembly is considerably more fragmented at extreme GC values. This technical bias also provides one possible explanation for the discrepancy between community composition estimates by Illumina and 454 sequencing in the original study (Shakya et al., 2013).

We also observe that the SSU rRNA genes have a higher average coverage and more moderate GC composition than the rest of the metagenome (**Figure 5a**), as many microbial genomes have multiple copies of the rRNA operon per genome, and the sequence conservation of the rRNA genes is relatively high compared to most protein-coding genes. This could make it difficult to bin a genome together with its corresponding rRNA operon(s) when relying only on coverage or sequence composition binning methods.

Automated binning with MetaBAT yielded many fragmentary bins. A total of 146 bins were predicted, however, not all contigs were assigned to a bin, particularly those which are short (<1000 bp), but surprisingly also some relatively large, high-coverage contigs (**Figure 5b**). Given that the community should contain only 64 strains, most bins are probably incomplete. A simple taxonomic annotation by taking the best Blastn hit to the NCBI nt database initially appeared to yield a similarly inflated diversity estimate: 403 species in 76 orders and 28 phyla. However, only 58 of those species assignments account for more than 1 Mbp of sequence each, a number which is more consistent with the known diversity in the AB mock community.

The genome of the archaeon *Nanoarchaeum equitans* assembled into a single contig of 474 kb, close to the published value of 491 kb (Waters et al., 2003). However, because MetaBAT defines genome bins as clusters of contigs, this individual contig was not assigned to a bin (**Figure 5b**). With the visualization in gbtools, this fact can be immediately recognized, and so the *Nanoarchaeum* contig was manually extracted. CheckM evaluates its completeness at 73.1% (**Table 3**), but this is attributable to the highly reduced nature of this genome.

**Table 3:** Summary statistics of the curated bins from the Archaea-Bacteria metagenome assembly.

Bin	Affiliation (Amphora2)^∗^	No. MetaBAT bins combined	Length (Mb)	Contigs	Amphora2 markers		CheckM completeness(%)	CheckM contamination(%)
					Total	Single-copy		
-	*Nanoarchaeum equitans*	0	0.474	1	86	84	73.1	0
23	*Haloferax volcanii*	4	5.17	474	134	108	100	28.2
28	*Methanosarcina acetivorans*	3	5.85	369	106	100	99.8	6.24
35	*Zymomonas mobilis*	1	2.00	86	32	30	99.8	0.92
13	*Chloroflexus* sp. Y-400-fl	1	4.98	141	31	31	99.7	0
10	*Wolinella succinogenes*	2	2.02	29	30	30	99.4	0.42
2	*Aciduliprofundum boonei*	3	1.38	23	104	101	99.2	0
32	*Nostoc* sp. PCC 7120	1	6.97	142	31	31	99.2	0
18	Igni*coccus hospitalis*	2	5.13	421	115	113	98.7	35.4
20	*Dictyoglomus turgidum*	2	1.47	14	31	31	98.3	0
38	*Thermus thermophilus*	3	1.95	59	29	29	98.1	0
7	*Archaeoglobus fulgidus*	5	2.05	37	108	101	98.0	0
44	*Akkermania muciniphila*	2	2.58	31	32	32	97.3	0
31	*Nitrosomonas europaea*	3	2.27	55	31	31	97.1	0.26
36	*Treponema denticola*	3	2.64	43	31	31	95.2	0
24	*Herpetosiphon aurantiacus*	1	5.68	67	31	31	94.6	0.91
19	*Geobacter sulfurreducens*	2	2.71	19	31	31	89.0	0
22	*Gemmatimonas aurantiaca*	1	2.01	6	31	31	87.9	0
15	*Deinococcus radiodurans*	3	2.48	168	31	29	79.3	0.21
1	*Acidobacterium capsulatum*	3	2.91	30	31	31	77.8	0.17
33	*Rhodopirellula baltica*	7	4.92	60	31	31	74.3	0
4	*Shewanella baltica*	2	2.04	458	35	29	50.0	0.92

*Majority consensus, if more than one species assignment.

**FIGURE 6:**
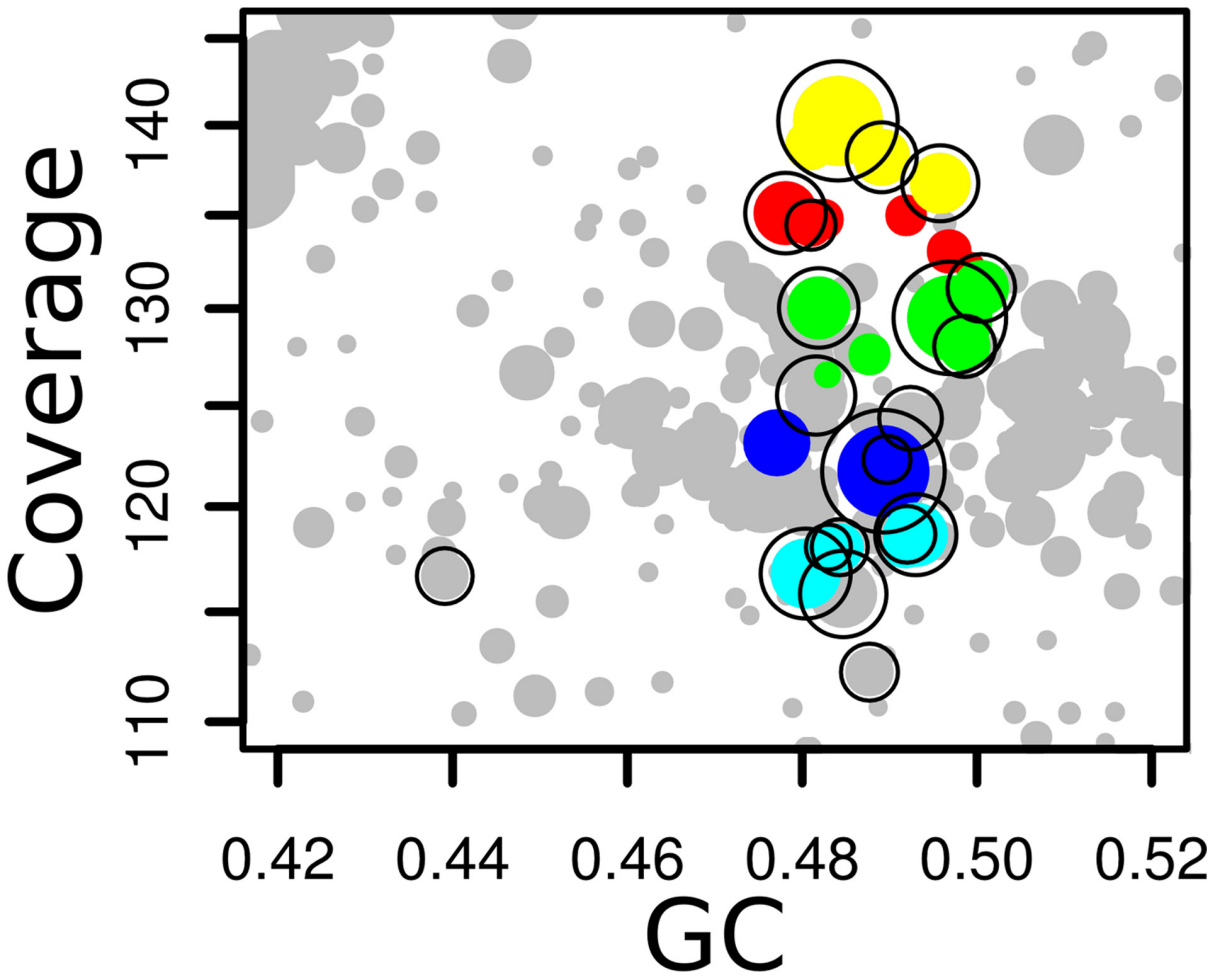
**Detail of coverage-GC plots for the synthetic AB metagenome, showing how MetaBAT bins that have Amphora2 taxonomic markers assigned to Archaeoglobales were merged to a single bin.** Colors – five individual MetaBAT bins that were merged; black outlines – contigs containing Archaeoglobales marker genes.

As with the *Olavius* example, we can combine different annotations and binning tools to produce curated bins with higher completeness. We used the conserved single-copy Amphora2 marker genes to identify which MetaBAT bins belong to the same taxa and should be merged. Unlike the previous example, it is impractical to do this individually for each bin. Instead gbtools has functions that can operate on and compare sets of bins. A list of new bins was created with the function binsFromMarkers() from the metagenome gbt object; each bin contains contigs that have Amphora2 markers with the same taxonomic annotation at the level of order. The MetaBAT bins were then merged into these new bins, with the function mergeOverlapBins(). The resulting 44 merged bins each contain one or more of the original MetaBAT bins, all of which have Amphora2 markers belonging to the same taxon. An example of such a merged bin is shown in **Figure 6**. The summary statistics of these bins were tabulated with summaryLOB(). Those merged bins that have at least 90% of the Amphora2 marker genes in single-copy (for the Bacteria marker set, 28 of 31, for Archaea, 94 of 106) were regarded as most likely to represent single genomes. This subset of 22 merged bins was exported, and checked for completeness with CheckM. The evaluation showed that 16 of the 22 had >90% completeness, and 13 of those 16 had contamination of <1% (**Table 3**). This shows that a relatively straightforward visualization-aided curation can already produce usable draft genomes from a complex sample.

## Conclusion

We show that hands-on exploration of data is not replaced by automated statistical methods for genome binning from metagenomic assemblies. Proper visualizations can suggest what automated methods to apply, and in return can be used to check the results of such analyses afterward. We offer gbtools to the community as a tool for this task that simplifies repetitive actions and lets users rapidly plot and manipulate their data. What distinguishes gbtools from existing software for metagenomic binning is that it is primarily a visualization tool which does not rely on any particular binning method. It is extensible, for users who may want to implement their own functions, and can be used with third-party tools for mapping and marker-gene annotation.

## Author Contributions

BS wrote the software. BS and HG tested the software, planned the manuscript, and wrote the manuscript.

## Conflict of Interest Statement

The authors declare that the research was conducted in the absence of any commercial or financial relationships that could be construed as a potential conflict of interest.

## Footnotes

1 http://genome.jgi.doe.gov/OlaalgELextract2/

2 http://genome.jgi.doe.gov/OlaalgELextract1_2/

3 http://www.ncbi.nlm.nih.gov/sra
